# Polymyxin Derivatives that Sensitize Gram-Negative Bacteria to Other Antibiotics

**DOI:** 10.3390/molecules24020249

**Published:** 2019-01-11

**Authors:** Martti Vaara

**Affiliations:** Northern Antibiotics, Espoo, Finland and Department of Bacteriology and Immunology, Helsinki University Medical School, Helsinki, Finland; martti.vaara@northernantibiotics.com; Tel.: +358-50-355-0822

**Keywords:** polymyxin B nonapeptide (PMBN), NAB7061, SPR741/NAB741, Enterobacteriaceae, *Acinetobacter baumannii*, *Pseudomonas aeruginosa*, synergism, permeabilizers, clinical phase 1 study

## Abstract

Polymyxins (polymyxin B (PMB) and polymyxin E (colistin)) are cyclic lipodecapeptide antibiotics, highly basic due to five free amino groups, and rapidly bactericidal against Gram-negative bacteria, such as the majority of Enterobacteriaceae as well as *Acinetobacter baumannii* and *Pseudomonas aeruginosa*. Their clinical use was abandoned in the 1960s because of nephrotoxicity and because better-tolerated drugs belonging to other antibiotic classes were introduced. Now, due to the global dissemination of extremely-drug resistant Gram-negative bacterial strains, polymyxins have resurged as the last-line drugs against those strains. Novel derivatives that are less toxic and/or more effective at tolerable doses are currently under preclinical development and their properties have recently been described in several extensive reviews. Other derivatives lack any direct bactericidal activity but damage the outermost permeability barrier, the outer membrane, of the target bacteria and make it more permeable to many other antibiotics. This review describes the properties of three thus far best-characterized “permeabilizer” derivatives, i.e., the classic permeabilizer polymyxin B nonapeptide (PMBN), NAB7061, and SPR741/NAB741, a compound that recently successfully passed the clinical phase 1. Also, a few other permeabilizer compounds are brought up.

## 1. Introduction

**Figure 1:**
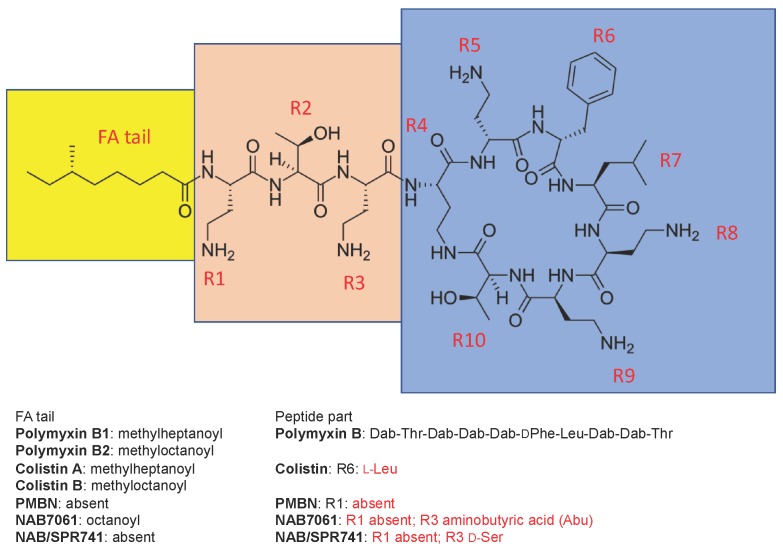
The structure of polymyxin B1 and several compounds structurally related to it. The fatty acyl tail of polymyxin B is highlighted with yellow, the linear “panhandle” part (i.e., residues R1-R3) with pink, and the cyclic heptapeptide part (i.e., residues R4-R10) with blue. Amino acid residues of colistin, PMBN, NAB7061, and NAB741/SPR741 that differ from those in polymyxin B are shown in red.

Polymyxins (polymyxin B (PMB) and polymyxin E (colistin)) are cyclic lipodecapeptide antibiotics, highly basic due to five free amino groups, and quite effective against Gram-negative bacteria such as the majority of Enterobacteriaceae as well as *Acinetobacter baumannii* and *Pseudomonas aeruginosa*. All members of the polymyxin class contain a cyclic heptapeptide core, linked to a linear tripeptide “panhandle” with an *N*-terminal fatty acyl moiety (Figure 1). The discovery of polymyxins was published independently by three laboratories in 1947 [1,2,3]. In 1948, a Washington D.C. newspaper [4] wrote “Time will tell, however, whether polymyxin will work in human beings. After all, many of the wonder drugs have reached this stage only to fail when put to the acid test”.

The clinical use of polymyxins was abandoned in the 1960s because of nephrotoxicity and because better-tolerated drugs belonging to other antibiotic classes were introduced. Now, due to the global dissemination of extremely-drug-resistant Gram-negative bacterial strains, polymyxins have resurged as the last-line drugs against those strains [5,6,7,8,9]. Amongst the alternatives, tigecycline suffers from low serum and urine levels and ceftazidime-avibactam from the emerging resistance and because it is not effective against carbapenemases belonging to class B [5,8,9].

However, even the new role of polymyxins is now in jeopardy. The recent appearance of mobile colistin resistance (*mcr*) genes has elicited lots of alarms and publicity, but strains resistant to polymyxins by other mechanisms, such as those with altered *mgrB*, display high-level resistance and are truly worrisome [10,11,12,13]. In the recent SENTRY study (isolates collected worldwide in 2017), the frequency of colistin resistance was still very rare; in *K. pneumoniae* (*n* = 3753) only 0.4% and in *E. coli* (*n* = 7397) only 0.3% [14]. On the other hand, amongst the global isolates of carbapenemase-producing *K. pneumoniae* (*n* = 1703) and *E. coli* (*n* = 407) collected during the SMART program in 2015–2016, the prevalence of colistin resistance was 21% and 14%, respectively [15]. In 2015, the European Antimicrobial Surveillance Network (EARS-Net) registered 33,100 deaths due to infections caused by antibiotic-resistant bacteria. Out of these, 6.8% was caused by colistin-resistant *E. coli* or *K. pneumoniae* and 0.5% by colistin-resistant *A. baumannii* or *P. aeruginosa* [16].

Polymyxins act specifically on Gram-negative bacteria and are rapidly bactericidal, while Gram-positive bacteria, eukaryotic microbes and mammalian cells are typically unaffected. They interact with anionic lipopolysaccharide (LPS) molecules, exclusively present in Gram-negative bacteria and located in the outer leaflet of their outer membrane (OM) [17,18]. The interaction damages the OM. The final and lethal action of polymyxins is a damage to the cytoplasmic membrane. As first shown by Vaara et al. in 1981, polymyxin-resistance is due to decoration of the lipid A part of LPS by phosphorylethanolamine and 4-amino-arabinose, both blocking the anionic binding sites of lipid A for polymyxin [19].

Polymyxin B and colistin are directly antibacterial. Novel derivatives of them (such as the FADDI compounds, CA824, MicuRx compounds, NAB739, and NAB815), all under preclinical development, might offer advantages such as enhanced efficacy in experimental infections, as recently reviewed by Vaara [20]. Other recent reviews on the development of novel derivatives include that of Rabanal and Cajal [21] and that of Brown and Dawson [22].

On the other hand, certain derivatives of polymyxins have lost their direct bactericidal activity but still damage the OM, restructure it, and weaken its function as a permeability barrier to many noxious agents, including antibiotics [17]. Accordingly, they act as “permeabilizers”, “sensitizers”, or “potentiators” (i.e., as agents that sensitize the bacteria to other antibiotics or potentiate the action of other antibiotics). The directly antibacterial polymyxins also exhibit potentiating activity at subinhibitory concentrations (see below) but the synergy indices are far lower because of the intrinsic activity of the direct-acting polymyxin derivative itself. Quite importantly, it has been shown, in the case of polymyxin B nonapeptide (PMBN, see below), that the stereochemical configuration is a determinant of the OM-damaging effect, since the enantiomer of PMBN lacks this activity [23].

This review sums up the properties of polymyxin derivatives that lack any notably direct antibacterial activity but sensitize the bacteria to other antibiotics.

## 2. Polymyxin B Nonapeptide (PMBN)

### 2.1. Synergism

Polymyxin derivatives that lack a fatty acid tail (i.e., des-fatty acyl derivatives) are significantly less active than polymyxin B against species such as *E. coli* and *K. pneumoniae*. On the other hand, they still possess the OM-permeabilizing activity and, hence, act as permeabilizers that allow enhanced entry of other antibiotics across the OM [17].

The classic permeabilizer, polymyxin B nonapeptide (PMBN, Figure 1) was discovered by Vaara and Vaara, published in 1983, and its properties have been extensively reviewed [17]. PMBN lacks the fatty acyl tail as well as the *N*-terminal diaminobutyryl (Dab) residue and is devoid of any direct antibacterial activity against enterobacterial species [24,25]. However, even at low concentrations (1–3 mg/L) it does widen the spectrum of many “anti-Gram-positive” antibiotics to cover Gram-negative bacteria, as well. These antibiotics include rifampin (decrease in the MIC, 100-fold) as well as erythromycin and fusidic acid (decrease in the MIC, 30-fold) [17]. Target organisms include *E. coli*, *K. pneumoniae*, several other enterobacterial species, and *P. aeruginosa*.

### 2.2. Efficacy

The combination of PMBN with erythromycin, as well as with novobiocin, administered intraperitoneally, protected mice infected with *K. pneumoniae* or *P. aeruginosa* in conditions where none of the agents alone was effective [26].

### 2.3. Toxicity

PMBN was shown to be 15 times less toxic than polymyxin B in an acute-toxicity assay in mice, 25 times less active in releasing histamine from rat mast cells, approximately 100 times less toxic in a eukaryotic cytotoxicity assay and approximately 150 times less active in causing neuromuscular blockade [17]. PMBN elicited no nephrotoxicity in a 23-day dog study and in a 29-day rat study at doses where polymyxin B did so [27]. Contemporary studies have confirmed the reduced nephrotoxic potential of PMBN. In vitro, PMBN was >50-fold less cytotoxic than polymyxin B toward human kidney proximal tubuli cell line HK-2 [28]. Furthermore, polymyxin B1 and colistin preferentially accumulate in the renal cortical region, while PMBN is more uniformly distributed throughout the kidney [29]. In a 7-day cynomolgus monkey study, PMBN at the daily dose of 60 mg/kg was less nephrotoxic than polymyxin B at the daily dose of 12 mg/kg [30].

### 2.4. Past and Contemporary Use

PMBN has been widely exploited as a useful tool to increase the permeability of the OM in various in vitro studies, including those dealing with the discovery and development of novel antibacterial drugs. A selected list of such studies conducted in years before 1992 can be found in the review by Vaara published in 1992 [17]. Thereafter, at least 16 studies on antibiotic development [31,32,33,34,35,36,37,38,39,40,41,42,43,44,45,46] and more than a dozen studies on bacterial molecular and cellular biology (eight representatives picked up here [47,48,49,50,51,52,53,54]) have used PMBN as a tool.

## 3. NAB7061

NAB7061 possesses the same heptapeptide core as polymyxin B, but its side chain consists of octanoyl-threonyl-aminobutyryl (Figure 1). As a consequence, it carries three positive charges only [55]. It lacks any notable direct antibacterial activity. However, at 4 µg/mL, it decreased the MIC of rifampin for *E. coli* (11 strains), other polymyxin-susceptible Enterobacteriaceae (12 strains), and *A. baumannii* (three strains) by factors of 85–750, 10–2,000, and 25–125, respectively [55]. With clarithromycin, the corresponding decreases in MIC were 90->750, 10–1000, and 40–100-fold, respectively. Furthermore, NAB7061 decreased the MIC of rifampin and clarithromycin for the polymyxin-resistant *K. pneumoniae* strain CL5762B by factors of 24 and 12, respectively [56]. The potentiating activity of NAB7061 has been verified in vivo in an *E. coli* peritoneal infection model in mice [57]. In contrast, neither NAB7061 or erythromycin alone was effective. After a single intravenous dose of 1 mg/kg to rats, the urinary recovery rate for NAB7061 was 7% [58]. When compared to that of colistin sulfate, the renal clearance of NAB7061 was approximately 30-fold higher. Several other structurally related sensitizing compounds have been described by Vaara et al., in 2008 [55,59]. NAB7061 has been shown to potentiate the action of several novel protein translocase SecA inhibitors that are excluded by the intact OM [43].

## 4. NAB741/SPR741

NAB741, discovered at Northern Antibiotics Ltd. (Espoo, Finland) by Vaara et al. and published in 2010 [18,60], possesses the same heptapeptide core as polymyxin B, but its side chain consists of acetyl-threonyl-D-serinyl (Figure 1). Accordingly, and as NAB7061, it carries only three positive charges, whereas the old polymyxins carry five. Furthermore, it lacks the hydrophobic fatty acyl tail present in the old polymyxins [18,60]. In 2015, Spero Therapeutics (Cambridge, MA, USA) in-licensed NAB741 and renamed it as SPR741. Thereafter, SPR741 has been under very extensive development, as evidenced for instance as the number of posters in the ASM Microbe conferences (32 posters in 2016–2018). In December 2016, SPR741 entered the clinical phase 1 study. In October 2017, Spero announced that the study yielded positive pharmacokinetic and tolerability data [61].

### 4.1. Mode of Action

Atomic force microscopy revealed that SPR741 causes undulations (approx. 10 nm) and breaks (approx. 20 nm) in the OM of *E. coli* [62]. No substantial impact was found on the cytoplasmic membrane, as assessed by the method utilizing 3,3′-dipropylthiadicarbocyanine iodine (DISC_3_(5)) [62]. In contrast to polymyxin B, SPR741 did not release ATP from the cells [63]. Two electron microscopy studies showed that SPR741 induces the formation of finger-like projections in the OM as well as blebbing of parts of the OM into medium [64,65]. These effects have previously been shown for polymyxin and PMBN [17].

### 4.2. Synergism

As shown by Vaara et al., in 2010 [61], NAB741 is notably synergistic with rifampin and clarithromycin against *E. coli, K. pneumoniae, E. cloacae*, and *A. baumannii.* The potentiation factors against these bacteria, as defined as the fold reduction in the MIC in the presence of 4 mg/L of SPR741 (relative to the MIC in the absence of SPR741) ranged from >2000 to 16 for rifampin and from 340 to 6 for clarithromycin. Marked synergism was also found with azithromycin, mupirocin, fusidic acid, and vancomycin against most of these bacterial species [61].

These findings were later corroborated by Corbett et al., in 2017 [66]. Out of 35 antibiotics tested, the MICs of eight were reduced 32 to 8000-fold against *E. coli* and *K. pneumoniae* in the presence of SPR741. The antibiotics were rifampin, four macrolides/ketolides (clarithromycin, azithromycin, erythromycin, telithromycin), the pleuromutilin antibiotic retapamulin, fusidic acid, and mupirocin. Corbett et al. showed that the potentiation factor against *E. coli* ATCC 25922 in the presence of 8 mg/L of SPR741 was 8000 for rifampin and 4000 for clarithromycin. Furthermore, SPR741 reduced the MIC of rifampin, clarithromycin, erythromycin, retapamulin, and fusidic acid for *A. baumannii* by >32 fold [66]. Amongst these antibiotics, retapamulin deserves a special interest, since it is a surrogate of lefamulin [67], an “anti-Gram-positive” antibiotic currently in late clinical development as a drug administered both intravenously and orally [68].

Other studies give further support to the original studies by Vaara et al., too. Hackel et al. showed strong synergism between SPR741 and rifampin, as well as with clarithromycin against recent clinical isolates *of E. coli*, *K. pneumoniae*, and *A. baumannii* [69]. Mendes et al. showed that SPR741 increases the activity of rifampin and clarithromycin against Enterobactericeae and *A. baumannii*, including strains displaying the MDR phenotype [70]. The potentiation was more marked for SPR741 plus rifampin, except against the MDR strains of *K. pneumoniae*. Their subsequent study revealed that the MIC_90_ values (the MICs for ≥90% of the strains) of clarithromycin in the presence of 8 mg/L of SPR741 were for *E. coli* (*n* = 178), *K. pneumoniae* (*n* = 115) and *E. cloacae* (*n* = 111), 2, 8, and 1 mg/L, respectively [71]. Both in *E. coli* and *K. pneumoniae*, four strains carried acquired resistance elements such as *ermB*, efflux pump element *mrs*, or the macrolide phosphorylase gene *mphA* [71]. These strains were not susceptible to the combination of SPR741 and clarithromycin.

In a study exclusively focused on *A. baumannii*, Zurawski et al. showed that amongst the 29 extensively drug-resistant strains, 28 strains were susceptible to the combination of SPR741 and rifampin [72]. In the study comprising 100 isolates of *A. baumannii*, the addition of SPR741 at 8 mg/L reduced the MIC_90_ from 16 mg/L to 2 mg/L [73].

Because of the impressive magnitude of potentiation exhibited by rifampin and clarithromycin in combination with polymyxin derivatives, most of the studies have focused on them as potential partner antibiotics to SPR741. However, chromosomal resistance to rifampicin, mediated by *rpoB*, could be expected to develop as easily in Gram-negative bacteria as has been experienced in Gram-positive bacteria. Furthermore, many MDR strains of Gram-negative bacteria have already been shown to possess resistant determinants that confer resistance to macrolide antibiotics (see above). Accordingly, these two antibiotics may not prove to be the partners of choice in clinical use.

Regarding potential partnering with betalactams, the MDR strains are generally resistant to piperacillin-tazobactam (TZP), ceftazidime and other betalactams. At the fixed concentration of 8 mg/L, SPR741 decreased the MICs of TZP-susceptible strains of *E. coli*, *K. pneumoniae*, and *E. cloacae* by a factor of 4–48 and that of TZP-resistant strains by a factor of ≥16 [74]. Quite similar results were obtained from another laboratory as well as from studies in which SPR741 was combined with ceftazidime, temocillin, or mecillinam [75,76,77]. Whether the synergism is due, at least in part, to the potential SPR741-induced leakage of the periplasmic betalactamases from the bacterial periplasm into the surrounding medium, has not yet been studied. PMBN is known to release periplasmic betalactamase as well as other periplasmic proteins into the medium [17]. No synergism was found between SPR741 and ceftazidime against anaerobic Gram-negative bacteria including *Bacteroides* spp., *Fusobacterium* spp., *Porphyromonas* spp., and *Prevotella* spp. [78].

Amongst the newer drugs under development against Gram-positive bacteria, two groups of non-quinolone-structured gyrase inhibitors are notably synergistic with SPR741. Both groups inhibit the *gyrB* gyrase and the *parE* topoisomerase IV. The ureabenzimidazole compounds SPR719 and SPR720 (in-licensed by Spero from Vertex in 2016) as well as the pyridylurea compounds SPR750 and SPR751 (in-licensed by Spero from Biota in 2016) are inactive against Gram-negative bacteria but in the presence of SPR741 at 8 mg/L their MICs for Enterobacteriaceae and *A. baumannii* are notably low (modal MICs, below 1 mg/L) [79,80,81,82,83]. Whether these drugs will be useful partners with SPR741 in clinical settings, will be seen. Other interesting compounds include the lantibiotic NAI-107, consisting of 23 amino acyl residues. It has been shown to be very synergistic with polymyxin B against Enterobacteriaceae and *A. baumannii* [84].

Other partner candidates will certainly include the tetracycline derivative minocycline. PMBN has long ago been shown to increase its entry to the cell [85]. However, since the bacteraemic Gram-negative infections often originate from complicated urinary tract infections (cUTI), it should be noted that tetracyclines are not very well excreted into urine. Still, other partners may include the streptogramin combination quinupristin–dalfopristin, as previously shown to be synergistic with NAB7061 [55] and with SPR741 [66]. Regarding other “old agents”, SPR741 reduces at 8 mg/L the MIC of fosfomycin for *E. coli* by a factor of 32 and at 4 mg/L by a factor of 16 [66].

In screening for any novel drugs under development that might be potential partners for SPR741, one should note that the desirable pharmaceutical formulation should be intended for intravenous (IV) use, because SPR741 is administered intravenously. Naturally, if the potential partner is very well absorbed orally, it might be useful as well. The entire concept of a sensitizer compound and its partner antibiotic has lots of challenges on its road to become clinically relevant (see Concluding Remarks below).

### 4.3. Efficacy in Experimental Infections

In the *K. pneumoniae* murine model of urinary tract infection, a combination of SPR741 at the dose of 10 mg/kg every 8 h (thrice a day, TID) and rifampin at the dose of 4 mg/kg TID was very effective in decreasing the bacterial load in the kidneys, whereas monotherapies with either agent alone were ineffective [86]. SPR741 at the dose of 10 mg/kg TID subcutaneously plus SPR720 (see above) at the daily dose of 60 mg/L orally was effective in decreasing the kidney bacterial load of carbapenem-resistant *K. pneumoniae* while both agents alone lacked any efficacy [87].

In the *A. baumannii* murine lung model, the combination of SPR741 at 10 mg/kg TID plus clarithromycin at 100 mg/kg once daily decreased the bacterial load by 1.15 log_10_ from the pretreatment [88]. This is remarkable, since the surfactants of the lung alveolar fluid may inactivate antibiotics as they do inactivate daptomycin. The report also states that SPR741 was effective in the pneumonia model following infection with *E. cloacae* expressing *bla*_KPC_ [88]. In line with these findings, SPR741 at the dose of 40 mg/kg every 12 h (twice a day, BID) plus rifampin at the dose of 5 mg/kg BID were therapeutic in an *A. baumannii* pneumonia model, whereas rifampin alone had only a modest efficacy [72].

Strains of Enterobacteriaceae seldomly cause complicated skin and soft tissue infections (cSSI). However, the neutropenic murine thigh infection model (representing cSSI) is widely used in pharmacokinetic/pharmacodynamic (PK/PD) evaluations. Therefore, five publications describe the performance of SPR741 in the thigh infection model. The study by Stainton et al., [89] employed the murine model and strains of Enterobacteriaceae as the challenge organisms and evaluated the efficacy to be equivalent to human doses (human-simulated regimens [HSR]. They showed that SPR741 at the HSR dose of 400 mg TIB plus azithromycin at the HSR daily dose of 500 mg were effective in reducing the bacterial burden, provided that the challenge organism displayed an azithromycin MIC ≤ 16 mg/L in the absence of SPR741. Accordingly, and quite expectedly, the combination of SPR741 and azithromycin did not show any effect against most of the strains that have acquired macrolide-resistance elements such as *mphA*, *mphE*, *msr,* and *ermB* [89]. The papers by Warn et al., [88,90,91] show that the combinations of SPR741 with rifampin and with clarithromycin were highly effective at reducing the thigh burden of mice infected with *E. coli*, *K. pneumoniae* and *A. baumannii*. Against *E. cloacae*, the combination of SPR741 and rifampin was effective (clarithomycin was not tested) [91].

The neutropenic murine thigh infection studies have also included one in which the combined efficacy of SPR741 and SPR719 (see above) was assessed. Whereas both agents alone were ineffective, the combination of SPR741 at the daily dose of 180 mg/kg and SPR719 at the daily dose of 150 mg/kg decreased the bacterial load by approx. 4 log_10_ from the vehicle control [92]. However, it should be noted that the doses of both drugs were very high.

### 4.4. Preclinical Pharmacokinetic and Toxicology Studies

After a single intravenous dose of 1 mg/kg to rats, the urinary recovery rate for NAB741 was 51% [61]. When compared to that of colistin sulfate, the renal clearance of NAB741 was approximately 400 times higher.

The cytotoxicity of SPR741 towards proximal tubular kidney cell lines has previously been shown to be much lower than that of the old polymyxins [93]. Polymyxin B caused total (>85%) necrosis of the electroporated porcine renal proximal tubular LCGPK1 cells at 0.016 mM, whereas a 32-fold concentration of NAB741 was needed for the same effect [93].

As discussed previously [20], agents that are better-tolerated than the old polymyxins in proximal tubular kidney cell cultures (i.e., “two-dimensional cultures”) may turn out to be disappointingly toxic in animal studies. The use of microphysiological systems (MPS) or “organs on chips” that are three-dimensional organ cultures might be a better predictor [20]. In the MPS utilizing primary proximal tubular epithelial cells from donors, polymyxin B elicited injury responses such as shedding of kidney injury molecule-1 (KIM-1) and induction of heme oxygenase-1 (HMOX-1) [94]. At equimolar concentration, NAB741 had no effect. Furthermore, polymyxin B, but not NAB741, upregulated the biosynthesis of cholesterol. Finally, NAB741 was bound to cell membranes (POPC lipid nanodiscs) with an affinity eight-fold lower than that for polymyxin B [94].

A non-GLP 7-day cynomolgus monkey study indicated that SPR741 was at the dose of 60 mg/kg/day less nephrotoxic than polymyxin B at the dose of 12 mg/kg/day [95]. A GLP (good laboratory practice) study lasting for 14 days indicated that the monkeys tolerated SPR741 at the dose of 40 mg/kg/day without any adverse effects [96]. The dose of 60 mg/kg/d caused mild to moderate nephrotoxicity that was fully reversible following a 28-day recovery period [96]. These results are in line with those obtained with the directly active derivatives NAB739 and NAB815 that carry three positive charges only. Both were better tolerated in cynomolgus studies than polymyxin B [20].

At the dose of 80 mg/kg/d, the plasma half-life of SPR741 was 1.4 h on day 1 and 2.0 h on day 14 [97]. SPR741 did not elicit in cynomolgus monkeys any cardiovascular or pulmonary effects at the single dose of 20 mg/kg. No neurological effects were found in the 14-day GLP study (80 mg/kg/day) [98]. SPR741 did not inhibit the hERG current at the highest concentration tested (300 mg/L) [98]. Furthermore, SPR741 was non-genotoxic in the battery of International Conference of Harmonization (ICH) tests [99]. No cytochrome P-mediated drug–drug interactions were found [100].

### 4.5. Phase 1 Clinical Trial

In the Phase I clinical trial, SPR741 was well-tolerated as a single intravenous dose of 800 mg (the highest dose tested) [101]. Subjects receiving SPR741 (maximum dose, 600 mg every 8 h intravenously, corresponding to the daily dose of 1800 mg) for 14 days tolerated the drug generally well, too [101]. However, three out of six subjects receiving the maximum dose showed mildly or moderately decreased clearance of creatinine. The plasma half-life at the dose of 600 mg every 8 h was approx. 2.3 h on day 1 and approx. 9 h on day 14 [102].

## 5. Concluding Remarks

Polymyxins increase the permeability of the OM to noxious agents but this action is in most instances masked by their direct bactericidal action which takes place at the very same concentrations. However, at subinhibitory concentrations many novel polymyxin derivatives do sensitize the target to antibiotics, in most studies to the model antibiotic rifampin [103,104,105,106]. The direct antibacterial drug NAB739 has been shown to sensitize at subinhibitory concentrations *A. baumannii* to rifampin, clarithromycin, fusidic acid, vancomycin, and meropenem [55].

Furthermore, polymyxin-like novel compounds, such as those under development by Rabanal et al., may act as sensitizers [107,108]. Sensitizers also include unacylated tridecapeptin A1 [109] and paenipeptins [110], both having structural relationships with polymyxins.

Linear peptides that carry cationic amino acyl residues integrated to a sequence of hydrophobic amino acyl residues (such as KFFKFFKFFK; K = lysyl, F = phenylalanyl) act synergistically with several antibiotics [111]. They may be rather toxic, even though no reports on their tolerability in animal models have not yet been published. Other permeabilizers that are not structurally related to polymyxins include those listed by Vaara in 1992 [17] as well as the cationic steroid antibiotics (CSA) and oligo-acyl-lysyls (OAKs), as reviewed by Zabawa et al., in 2016 [112].

The “permeabilizer” concept, where a permeabilizer (such as SPR741) makes the OM of the target bacteria permeable to other agents (partner antibiotics), is scientifically meaningful. Further studies are needed to evaluate the efficacy and tolerability of the combination of SPR741 with other antibiotics in clinically relevant conditions. The existence and further development of chromosomal and acquired resistance against many of the potential partner antibiotics makes the choice of the final partner very difficult. Furthermore, if both the permeabilizer and the partner completely lack any direct antibacterial activity by themselves, Phase 2 clinical studies may be quite problematic to justify in regulatory terms. One of the concrete alternatives for a partner antibiotic to SPR741 in a potential Phase 2 or Phase 2/3 clinical study would be a wide-spectrum betalactam (piperacillin-tazobactam, ceftatzidime, aztreonam). They have notable activity even against strains that have been labelled as nonsusceptible by the laboratory standards. As presented above, SPR41 would potentiate their action against the target. Whether Spero is going to take SPR741 into the clinical Phase 2 has not been announced.

Regarding the emerging polymyxin-resistant (PMR) strains (see Introduction), Tascini et al. showed in 2013 that there is notable synergism with colistin and rifampin against the PMR strains of *Klebsiella pneumoniae* that produce carbapenemase [113]. Very recently (2018), two publications have highlighted the potential of polymyxin-based combinations against PMR strains in a detailed way. MacNair et al. showed that colistin is notably synergistic in vitro with rifampin, the macrolide antibiotic clarithromycin, minocycline, and novobiocin against *mcr* strains [114]. Brennan-Krohn et al. described the synergism with colistin with rifampin, the macrolide antibiotic azithromycin, minocycline, fusidic acid, linezolid, and several other drugs against *mcr* and other PMR strains [115]. Interestingly, also the novel polymyxin derivative NAB739 [18,20,55] is synergistic with rifampin and retapamulin against PMR strains [116]. Accordingly, polymyxins that are directly bactericidal against wild-type bacteria still possess the OM-permeabilizing activity against strains that are resistant to the killing action of polymyxins. This finding adds new hope in the battle against extremely drug-resistant Gram-negatives.
